# RIP3 induces ischemic neuronal DNA degradation and programmed necrosis in rat *via* AIF

**DOI:** 10.1038/srep29362

**Published:** 2016-07-05

**Authors:** Yang Xu, Jingye Wang, Xinghui Song, Lindi Qu, Ruili Wei, Fangping He, Kai Wang, Benyan Luo

**Affiliations:** 1 Department of Neurology, Brain Medical Centre, First Affiliated Hospital, Zhejiang University School of Medicine, 89 Qingchun Road, Hangzhou 310003, China; 2 Department of Neurology, First Affiliated Hospital, Anhui Medical University, 218 Jixi Road, Hefei 230022, China; 3 Facility for Biochemistry and Molecular medicine Core Facilities, Zhejiang University School of Medicine, 866 Yuhangtang Road, Hangzhou 310058, China

## Abstract

We have reported that nuclear translocation of Receptor-interacting protein 3 (RIP3) involves in neuronal programmed necrosis after 20-min global cerebral ischemia/reperfusion (I/R) injury. Herein, the underlying mechanisms and the nuclear role of RIP3 were investigated further. The necroptosis inhibitor necrostatin-1 (Nec-1), the autophagy inhibitor 3-methyladenine (3-MA), and the caspase-3 inhibitor acetyl-L-aspartyl-L-methionyl-L-glutaminyl-L-aspart-1-al (Ac-DMQD-CHO) were administered intracerebroventricularly 1 h before ischemia. Protein expression, location and interaction was determined by western blot, immunofluorescence or immunoprecipitation. Most CA1 neuronal death induced by 20-min global cerebral I/R injury was TUNEL-positive. Neuronal death and rat mortality rates were greatly inhibited by Nec-1 and 3-MA pre-treatment, but not by Ac-DMQD-CHO. And no activation of caspase-3 was detected after I/R injury. Caspase-8 was expressed richly in GFAP-positive astrocytes and Iba-1-positive microglia, but was not detected in Neun-positive neurons. The nuclear translocation and co-localization of RIP3 and AIF, and their interaction were detected after I/R injury. These processes were inhibited by Nec-1 and 3-MA pre-treatment, but not by Ac-DMQD-CHO. The formation of an RIP3-AIF complex and its nuclear translocation are critical to ischemic neuronal DNA degradation and programmed necrosis. Neurons are more likely to enter the programmed necrosis signal pathway for the loss of caspase-8 suppression.

Programmed cell death (PCD) plays a prominent role in the processes of growth and development, as well as diseases. Based on morphological and biochemical features, PCD is classified into three types at present: apoptosis, programmed necrosis, and autophagic cell death^1^,^2^. Apoptosis, the first discovered form of PCD, is subdivided into two categories based on the molecules involved: caspase-dependent and caspase-independent pathways^3^. Most of the caspase-independent pathways act on mitochondria, and apoptosis-inducing factor (AIF) is considered to be the main effector, because its nuclear translocation occurs in nearly all caspase-independent PCD paradigms^4^,^5^. After a caspase-independent cell death insult, AIF is released from mitochondria and then translocates into the nucleus, where it participates in chromatin condensation and chromatinolysis^4^,^6^,^7^.

Programmed necrosis, also termed necroptosis, is another form of caspase-independent PCD. It was first identified by Degterev, who used tumor necrosis factor alpha and Fas ligand to induce cell death in different cell lines^8^. Necroptosis exhibits some of the morphological features of necrosis, but it can be controlled by many molecules. Formation of the receptor-interacting protein kinase 1 (RIP1) and RIP3 complexes (called necrosomes) is the key initiation step for necroptosis. And RIP3 has been found to be a molecular switch in the conversion to apoptosis and necroptosis, while RIP1 is reported not to be involved in all kinds of necroptosis^9^,^10^,^11^,^12^,^13^. DNA alkylating agents induce another kind of caspase-independent PCD known as programmed necrosis (also called parthanatos), in which AIF is the key molecule^4^,^6^,^7^.

In a previous study, we showed that 20-min global cerebral ischemia/reperfusion (I/R) injury induces hippocampal CA1 neuronal death by programmed necrosis^14^, the expression of RIP3 is upregulated, and it is translocated from the neuronal cytoplasm into the nucleus in this process; this has also been confirmed in studies of neuronal death after traumatic brain injury^15^,^16^. But the role of RIP3 in I/R injury-induced neuronal programmed necrosis and its mechanisms of action in the nucleus are unclear. In this study, we observed that RIP3 combined with apoptosis-inducing factor (AIF) and translocated into the nucleus after I/R injury. Further, the undetectable expression of caspase-8 suggested that neurons are more likely to undergo necroptosis.

## Results

### Pre-treatment with nec-1 and 3-MA inhibits the hippocampal CA1 neuronal death induced by global cerebral I/R injury

**Figure 1:**
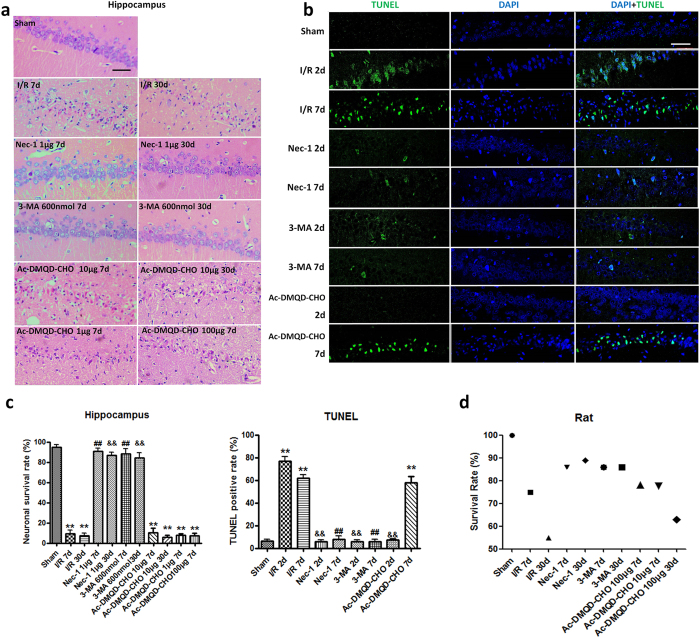
Protective effects of Nec-1 and 3-MA pre-treatment on neuronal death after 20-min global cerebral I/R injury. (**a**) Representative HE staining of hippocampal CA1 neurons at 7 and 30 days post-reperfusion in the sham, I/R, Nec-1, 3-MA, and Ac-DMQD-CHO groups. Normal pyramidal neurons showed round and pale nuclei, while dying or dead neurons exhibited pyknotic nuclei or a light-red staining profile. All photomicrographs are 400× (n = 6). (**b**) Representative micrographs of TUNEL staining (green) in the hippocampal CA1 region from the sham, I/R, Nec-1, 3-MA, and Ac-DMQD-CHO groups (n = 3). Scale bar = 50 μm. (**c**) Left panel was the neuronal survival rate in the hippocampal CA1 region at 7 and 30 days post-reperfusion. Right panel was TUNEL-positive rate in the CA1 region after I/R injury (**P < 0.01 *vs* sham group, ^##^*P* < 0.01 *vs* I/R 7d group, ^&&^*P* < 0.01 *vs* I/R 30d group in HE or I/R 2d group in TUNEL). (**d**) Rat mortality rate of each group.

To determine the protective effect of different signaling pathway inhibitors of necroptosis (Nec-1), autophagy (3-MA), or caspase-3 (Ac-DMQD-CHO), which were given 1 h before ischemia, the neuronal survival rate in the hippocampal CA1 region was evaluated. Morphologically, only 9.3 ± 1.3% and 7.4 ± 3.0% of the hippocampal CA1 neurons in the control group survived at 7 and 30 days, respectively, which demonstrated that almost all the neuronal death occurred within 7 days after reperfusion. The survival rates in the 1 μg, 10 μg, and 100 μg Ac-DMQD-CHO groups were 7.6 ± 0.8%, 10.1 ± 1.9%, and 7.4 ± 0.9% at 7 days post-reperfusion, while they were 90.9 ± 1.1% in the Nec-1 and 88.2 ± 2.0% in the 3-MA pre-treatment groups. And the protective effects of Nec-1 and 3-MA continued for 30 days, since the survival rates were 86.7 ± 1.3% and 84.1 ± 2.1% at 30 days post-reperfusion, respectively. Nevertheless, the survival rate in the 10 μg Ac-DMQD-CHO group was still 5.8 ± 0.7% at 30 days post-reperfusion. These results suggested that the protective effect of Nec-1 or 3-MA against hippocampal CA1 neuronal death is long-term action rather than just delaying death. However, 1 μg, 10 μg, and 100 μg Ac-DMQD-CHO had no protective effect, so 10 μg Ac-DMQD-CHO was used in the subsequent experiments (Fig. 1a,c). Corresponding to neuronal death, the rat mortality rate declined in the Nec-1 and 3-MA groups, but did not change in the Ac-DMQD-CHO group (Fig. 1d).

### Pre-treatment with Nec-1 and 3-MA prevent neuronal DNA cleavage

Neuronal DNA cleavage induced by transient global cerebral I/R injury was assessed by TUNEL staining at 2 and 7 days post-reperfusion (Fig. 1b). At 2 days, 76.5 ± 2.4% of CA1 neurons were positive for TUNEL staining and this decreased to 61.6 ± 1.8% while the fluorescence intensity increased and the nucleus appeared denser at 7 days. TUNEL-positive neurons in the CA1 region almost completely absent in the Nec-1 and 3-MA groups both at 2 and 7 days. No TUNEL-positive cells were observed at 2 days in the Ac-DMQD-CHO group, but the rate of TUNEL-positive staining was still 57.2 ± 2.8% at 7 days post-reperfusion and there was no difference between the Ac-DMQD-CHO and the I/R 7-days groups (Fig. 1c).

### Caspase-3 and caspase-8 are not involved in regulating hippocampal CA1 neuronal death induced by I/R injury

**Figure 2:**
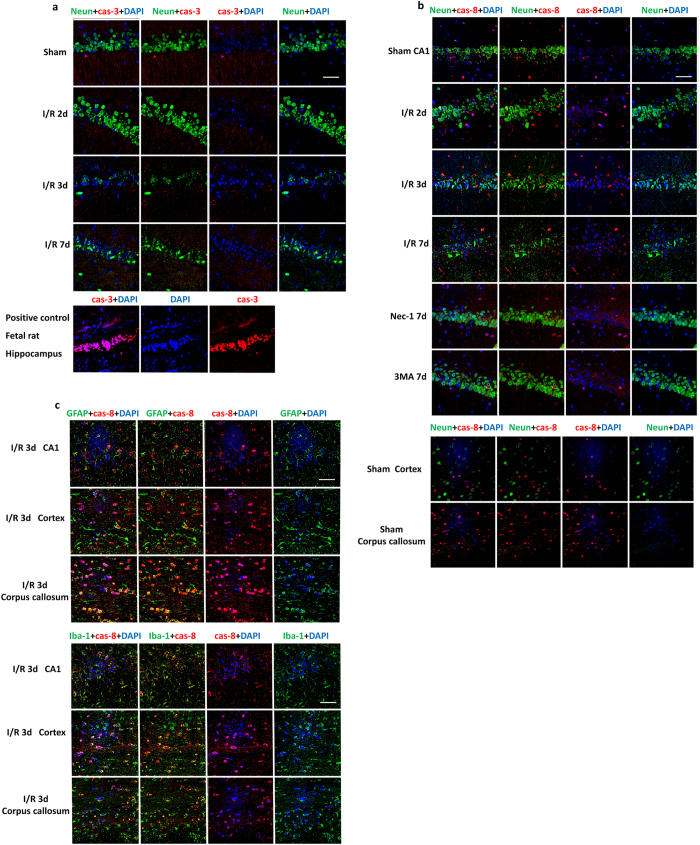
No activation of caspase-3 and no expression of caspase-8 in neurons after I/R injury. (**a**) Representative micrographs of cleaved caspase-3 in the hippocampal CA1 region. Many cells staining positive for cleaved caspase-3 (cas-3) occurred in the positive control, fetal rat brain. No positively-stained cells were detected in any of the sham, I/R 2d, I/R 3d, and I/R 7d groups. (**b**) Representative micrographs of caspase-8 (cas-8) immunofluorescent staining in the hippocampus, cortex and corpus callosum. No caspase-8-positive cells co-localized with Neun in any groups. The number of caspase-8-positive cells increased slightly after I/R injury, and this was inhibited by Nec-1 and 3-MA pre-treatment. (**c**) Micrographs of caspase-8 immunofluorescent staining with GFAP and Iba-1. Caspase-8 was expressed in GFAP-positive astrocytes and in Iba-1-positive microglia, and was especially rich in the corpus callosum (n = 3, scale bar = 50 μm).

Many cleaved caspase-3-positive cells were observed in the fetal rat brain, but none were detected at 2, 3, and 7 days post-reperfusion in the hippocampal CA1 region, suggesting that caspase-3 is not activated after I/R injury (Fig. 2a). Almost no caspase-8-positive cells co-labeled with Neun in the hippocampal CA1 region in the sham and I/R injury groups (Fig. 2b), and this was similar in the corpus callosum and cortex in the sham group, demonstrating that caspase-8 is not expressed in neurons in rat. Meanwhile, some cells with smaller nuclei were positive for caspase-8 (located both in the cytoplasm and mainly in the nucleus) in the hippocampus and cortex, and especially richly in the corpus callosum (Fig. 2b), suggesting that they might be glial cells. Besides, the number of caspase-8-positive cells increased slightly in the hippocampus after I/R injury, and decreased in the groups pretreated with Nec-1 and 3-MA. So, caspase-8 was co-stained with Iba-1 or GFAP antibody and IF was used to determine the type of cell expressing caspase-8 at 3 days post-reperfusion. Most GFAP-positive and Iba-1-positive cells were co-labeled by caspase-8, demonstrating that caspase-8 is expressed in astrocytes and microglia richly (Fig. 2c).

### Co-localization and nuclear translocation of RIP3 and AIF in hippocampal CA1 neurons after I/R injury

**Figure 3:**
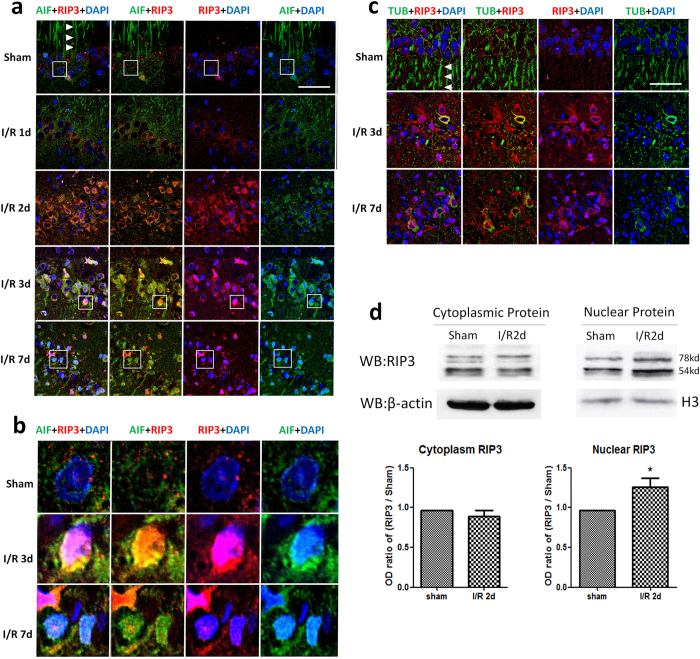
Nuclear translocation and co-localization of RIP3 and AIF after I/R injury. (**a**,**b**) Representative micrographs of AIF (green) and RIP3 (red) immunofluorescence. Photomicrographs in B are four times enlarged areas of the boxes in A. In the sham group, RIP3 was located mainly in the cytoplasm and AIF completely outlined the shapes of axons (arrowheads). RIP3 co-labeled with AIF not only in the perinuclear cytoplasm, but also in nuclei with condensed DAPI staining at 2 and 3 days post-reperfusion. At 7 days post-reperfusion, AIF fluorescence intensity remained high in the nucleus, while RIP3 weakened greatly. (**c**) Immunofluorescent localization of β-tubulin-III (green) and RIP3 (red) in hippocampal CA1 neurons in the sham and I/R groups. Complete axons were demonstrated by β-tubulin-III fluorescence in the sham group (arrowheads), and they disappeared after I/R injury. Unlike RIP3, no co-localization of β-tubulin-III fluorescence with DAPI was detected. (n = 3, scale bar = 50 μm). (**d**) Western blot analysis showing the nuclear translocation of RIP3 at 2 days post-reperfusion (n = 3, *P < 0.05 *vs* sham group).

Double immunofluorescent labeling was used to assess changes in the expression and localization of AIF and RIP3 in hippocampal CA1 neurons. In the sham group, the RIP3 fluorescence was located in the cytoplasm, predominantly around the nucleus. The AIF fluorescence was distributed both in the perinuclear cytoplasm and in axons; the latter was especially evident and axonal morphology was clearly revealed (arrowheads in Fig. 3a). The IF intensity of RIP3 showed a large increase in the perinuclear cytoplasm, and RIP3 IF labeling in pyknotic neuronal nuclei with condensed DAPI labeling was observed in most neurons at 3 days post-reperfusion. After I/R injury, the axonal distribution of AIF disappeared from day one post-reperfusion. Then, IF increased markedly in the cytoplasm close to the nuclear envelope at 2 days post-reperfusion, and was co-labeled with pyknotic neuronal nuclei in most neurons at 3 days post-reperfusion. Interestingly, IF co-labeling of RIP3 with AIF was detected not only in the perinuclear cytoplasm, but also in the nucleus. These observations demonstrated the synchronous translocation of RIP3 and AIF into the nucleus and the peripheral region of the nuclear envelope (Fig. 3a,b). Nuclear RIP3 levels decreased greatly in most neurons at 7 days post-reperfusion, while co-labeling of AIF with DAPI remained clear in many neurons (Fig. 3a,b). To further assess the changes of RIP3, cytoplasmic and nuclear proteins were extracted and quantified by Western blot. At 2 days post-reperfusion, there was no significant change of the cytoplasmic protein level of RIP3, while the nuclear protein level increased greatly, further confirming its nuclear translocation after I/R injury (Fig. 3d).

β-Tubulin-III, a microtubule element expressed exclusively in neuronal cytoplasm, is a good marker for displaying the morphology of axons (arrowheads in Fig. 3c). After I/R injury, the axonal distribution of β-tubulin-III disappeared just like AIF, which demonstrated the loss of axonal integrity. Unlike AIF, no co-localization of β-tubulin-III IF with DAPI and RIP3 was detected, suggesting that it does not translocate into the nucleus like RIP3 and AIF (Fig. 3c).

### Pre-treatment with Nec-1 and 3-MA inhibits the nuclear translocation and co-localization of RIP3 and AIF

**Figure 4:**
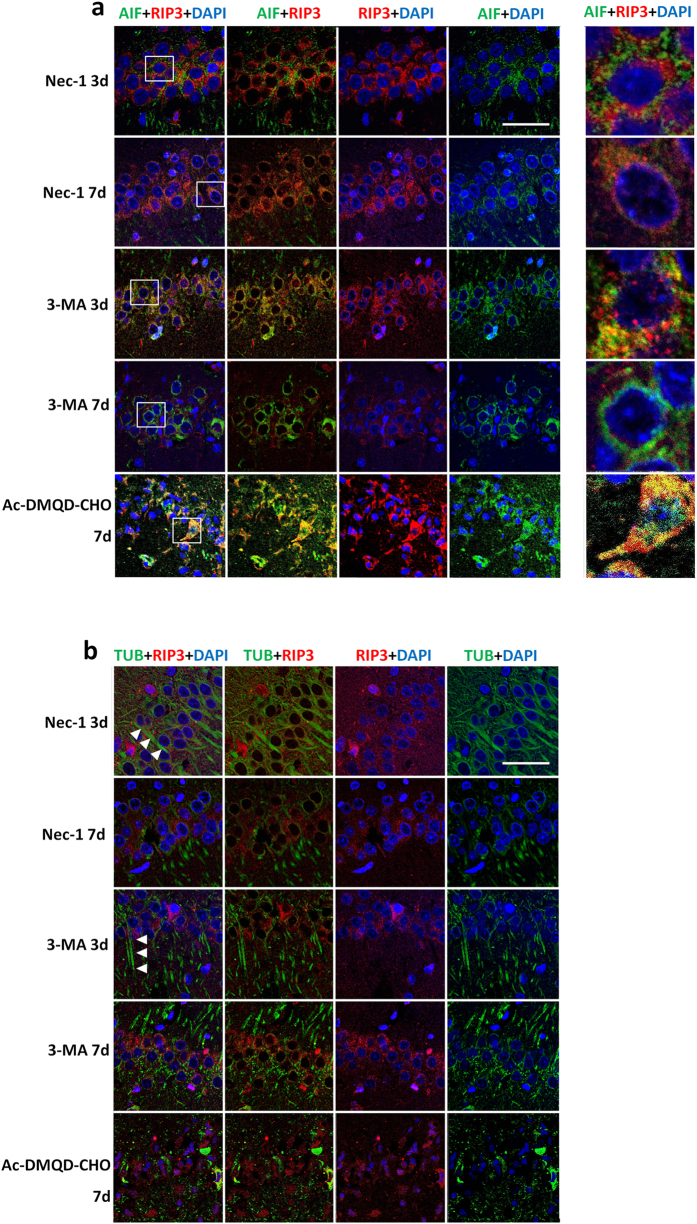
Pre-treatment with Nec-1 and 3-MA inhibits the co-localization and nuclear translocation of RIP3 and AIF. (**a**) Nec-1 and 3-MA pre-treatment inhibits the nuclear translocation and co-localization of RIP3 (red) and AIF (green) induced by I/R injury. Although there was no nuclear translocation of AIF, the cytoplasmic localization of AIF close to the nuclear envelope was also evident in the 3-MA group. Ac-DMQD-CHO did not prevent the overlap of RIP3 and AIF with nuclear shrinkage indicated by DAPI. Right panel: enlargements four times of neurons in the boxes in the left panel. (**b**) Nec-1 and 3-MA pre-treatment maintained the complete morphological localization of RIP3 (red) and β-tubulin-III (green) in hippocampal CA1 neurons. Ac-DMQD-CHO did not prevent the changes of RIP3 and the disintegration of β-tubulin-III (n = 3, scale bar = 50 μm).

The nuclear translocation and co-localization of AIF and RIP3 was prevented almost completely by Nec-1 and 3-MA pre-treatment (Fig. 4a), and we inferred that axons were not damaged from the axonal distribution of β-tubulin-III fluorescence in these two groups (Fig. 4b). Meanwhile, the axonal distribution of AIF was not intact, and the damage was more severe in the 3-MA group (Fig. 4a). Further, the increased cytoplasmic localization of AIF close to the nuclear envelope was also evident in the 3-MA group both at 3 and 7 days post-reperfusion, while it was inhibited in the Nec-1 group. Changes of RIP3 close to the nuclear envelope similar to AIF were not detectable in the Nec-1 and 3-MA groups (Fig. 4a). IF investigation showed that Ac-DMQD-CHO did not inhibit the nuclear translocation of AIF and RIP3, axonal integrity, and nuclear pyknosis (Fig. 4a,b). These results suggested that the nuclear translocation and co-localization of RIP3 and AIF induced by I/R injury are prevented by 3-MA, whereas the release of AIF from mitochondria is not inhibited by 3-MA. The changes of both RIP3 and AIF (except for the partial axonal distribution of AIF) were prevented by Nec-1, but not by Ac-DMQD-CHO.

### Pre-treatment with Nec-1 and 3-MA inhibits the interaction of RIP3 with RIP1 and AIF in the process of I/R injury

**Figure 5:**
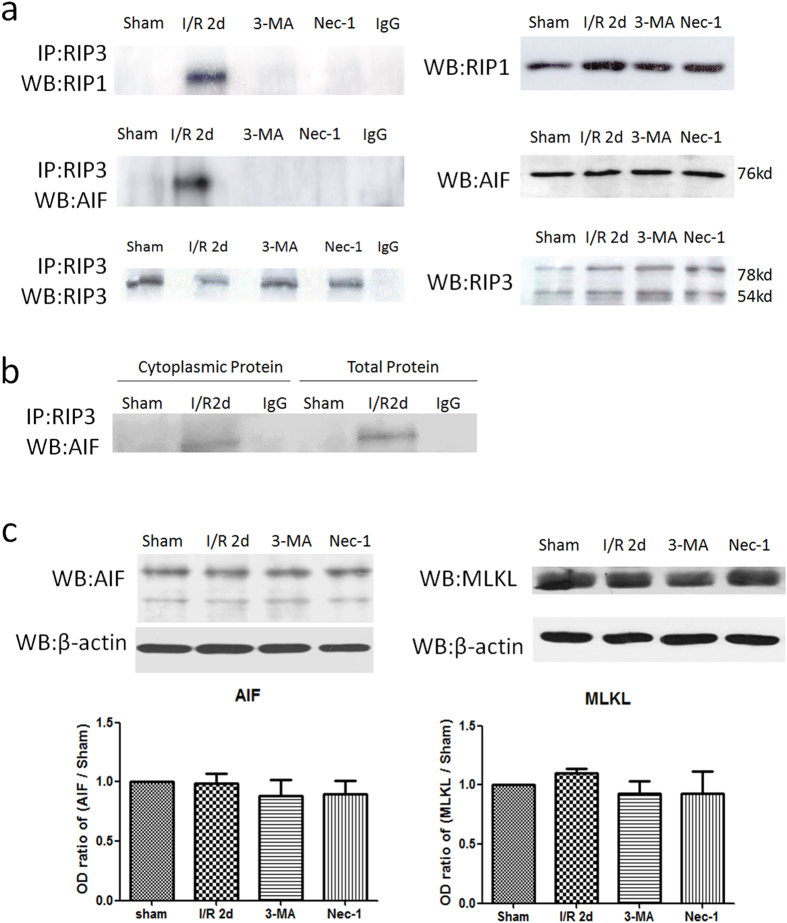
RIP3 interacted with RIP1 or AIF, and no expression changes of AIF and MLKL in the process of I/R injury. (**a**) Representative IP bands of RIP3 with RIP1 and AIF after I/R injury in total protein. No bands were detected in the sham group, while interactions of RIP3 with RIP1 and AIF occurred at 2 days post-reperfusion, and both were inhibited by Nec-1 and 3-MA (n = 3). Protein bands of RIP1, RIP3, and AIF in total protein solution detected by WB analysis in right panel (n = 3). (**b**) Interaction of RIP3 with AIF was found both in the total protein and cytoplasmic protein, suggesting that RIP3 interacts with AIF in the cytoplasm. (**c**) Representative WB bands of AIF and MLKL and relative OD ratios. AIF and MLKL expression did not significantly change during the process of I/R injury. (n = 3, **P < 0.01 *vs* sham group, ^##^*P* < 0.01 *vs* I/R 2d group).

We used IP to detect proteins interacting with RIP3 in the process of I/R injury. No IP bands of RIP3 with RIP1 and AIF were detected in the sham group, while clear bands suggested interactions of RIP3 with RIP1 and AIF at 2 days post-reperfusion, both of which were significantly inhibited by Nec-1 and 3-MA (Fig. 5a). To further determine whether RIP3 combined with AIF in the cytoplasm, total protein and cytoplasmic protein were extracted from CA1 tissue and assessed by IP. An RIP3-AIF interaction was found both in the total protein and cytoplasmic protein, suggesting that RIP3 interacts with AIF in the cytoplasm (Fig. 5b). These data suggested that RIP3 combines with AIF in the cytoplasm to form a complex after 20-min I/R injury and then translocate into the nucleus, while pre-treatment with Nec-1 and 3-MA inhibits their combination.

### No expression changes of AIF and MLKL

After I/R injury, no expression changes of AIF and MLKL were detected at 2 days post-reperfusion, and their levels were not influenced by Nec-1 and 3-MA pre-treatment (Fig. 5c).

## Discussion

The TUNEL assay, long used as a marker of apoptosis, is the classical method of detecting DNA fragmentation. It has been reported that programmed necrosis also shows TUNEL positivity, but the DNA degradation mechanisms are unclear^17^. In our 20-min global cerebral I/R injury model, most dying neurons in the CA1 region were also TUNEL-positive at 2 and 7 days post-reperfusion. And in a previous study using electron microscopy, we showed that this kind of neuronal death is programmed necrosis^14^. In apoptosis, DNA degradation is mainly mediated via two signal pathways: caspase-mediated type II apoptosis, and AIF-mediated type I apoptosis. So we assessed the changes of these molecules for DNA degradation patterns that are similar in programmed necrosis and apoptosis.

Ac-DMQD-CHO, Z-VAD-FMK, and Z-DEVD-FMK are commonly-used caspase-3 inhibitors. Since it has been reported that Z-DEVD-FMK also inhibits caspase-7 and Z-VAD-FMK also inhibits caspase-1 in addition to caspase-3 inhibition^18^,^19^, we used Ac-DMQD-CHO, a relatively specific inhibitor of caspase-3, to determine whether caspase-3 involved in this process. No activation of caspase-3 in neurons was detected in the hippocampal CA1 region in sham and I/R-injured rats. And further, when the caspase-3 inhibitor Ac-DMQD-CHO was given 1 h before ischemia, it did not show any protective effect against CA1 neuronal death except for a transient inhibition of TUNEL staining at 2 days post-reperfusion. Yasuhito *et al*. have also reported that CA1 pyramidal cell death induced by transient cerebral ischemia in rats is protected by Z-VAD-FMK, while Ac-DMQD-CHO has no such protective effect^19^. Caspase-8 is an endogenous inhibitor of the necroptosis signal by cleaving and inactivating RIP1 and RIP3. When it is knocked out or inhibited, the cell death pathway switches from apoptosis to necroptosis^13^,^20^,^21^. Using IF assays, we found no caspase-8 expression in any Neun-positive neurons in the hippocampus and cortex. However, it was expressed in GFAP-positive astrocytes and in Iba-1-positive microglia. Necroptosis is a form of PCD that occurs when caspases are inhibited or knocked out, and this is further supported by our observations in neuronal programmed necrosis induced by I/R injury. No detectable expression of caspase-8 was also observed both in the human neuroblastoma cells and rat cortical neurons. 24(S)-Hydroxycholesterol induce neuronal cell death by necroptosis, while 24(S)-Hydroxycholesterol-treated human T lymphoma cells with caspase-8 expression showed typical apoptotic features^22^. Similar lower expression levels of procaspase-8 have been found in necroptosis-prone glioma cells compared with the levels in other cancer cell types that undergo apoptosis^23^. These observations suggest that caspase-8 expression is an important factor that determines the death manner even under the same stimulus. Besides, caspase-8 was reported to be the key molecule involved in microglia activation, inflammation and neurotoxicity^24^,^25^. In our study, undetectable expression of caspase-8 in neurons suggested that neurons are more likely to enter the necroptosis signal pathway for the loss of its suppression; and its rich expression in microglia and astrocytes may be a factor to regulate their activation after cerebral I/R injury.

Chromatin condensation and DNA degradation triggered by AIF represent the most important caspase-independent apoptotic pathway. AIF was originally described as a mitochondrial inter-membrane protein, which translocates into the nucleus after apoptosis induction and triggers DNA degradation^4^,^6^. AIF is also involved in programmed necrosis in specific cell death pathways, including those activated by excitotoxins such as N-methyl-D-aspartate and glutamate, hypoxia-ischemia, DNA-alkylating agents, or growth factor deprivation^5^. Our WB analysis showed that the expression level of AIF in the hippocampal CA1 region did not change after I/R injury. However, it was released from mitochondria into the cytosol and translocated into the nucleus from 2 days post-reperfusion, and this was also clear at 7 days post-reperfusion. These results suggested that AIF is involved in the DNA degradation of CA1 neurons induced by global cerebral I/R injury.

Double immunofluorescence demonstrated that RIP3 and AIF co-localized not only in the perinuclear cytoplasm but also in the nucleus after I/R injury. For comparison, we used β-tubulin-III, a microtubule molecule expressed mainly in axons. β-Tubulin-III lost its axonal distribution like AIF and dispersed in the cytoplasm after I/R injury, demonstrating that the integrity of axons was destroyed. But β-tubulin-III did not translocate into nucleus like AIF and RIP3, suggesting that the nuclear translocation of AIF and RIP3 is not due to disruption of the nuclear membrane. The co-localization of RIP3 and AIF indicated that they interact after I/R injury, so we used immunoprecipitation to investigate this. As expected, formation of the RIP3-AIF complex increased greatly at 2 days post-reperfusion, with no IP bands for AIF detected in sham group. And then, we extracted cytoplasmic protein and further demonstrated that RIP3 combined with AIF in the cytoplasm. All the data suggested that the interaction of AIF with RIP3 and their nuclear translocation are key steps in the process of neuronal programmed necrosis induced by 20-min global cerebral I/R injury. Besides, the RIP3-RIP1 complex was also detected in our model at 2 days post-reperfusion. It has been reported that RIP1 and RIP3 is phosphorylated and activated in this process^12^. So RIP3 may be activated after I/R injury, interacts with AIF in the cytoplasm, and then is translocated into the nucleus.

Subsequently, we intracerebroventricularly administered the RIP1 kinase inhibitor Nec-1, the autophagy inhibitor 3-MA, and the caspase-3 inhibitor Ac-DMQD-CHO 1 h before ischemia to investigate their effects. The interaction of RIP3 with RIP1 and AIF, the nuclear translocation of RIP3 and AIF, the damage to axonal integrity, and the neuronal programmed necrosis induced by global cerebral I/R injury were inhibited by pre-treatment with both Nec-1 and 3-MA. However, there are some differences: the loss of axonal distribution of AIF and its accumulation in the perinuclear cytoplasm were also evident in the 3-MA pre-treatment group, but not so marked in the Nec-1 pre-treatment group, suggesting that the release of AIF from mitochondria is not completely inhibited by 3-MA. Ac-DMQD-CHO had no protective effect, not only on neuronal death but also on the changes of AIF and RIP3. These results demonstrated that: (i) the nuclear translocation of AIF and RIP3 is critical to neuronal programmed necrosis; (ii) neurons do not proceed to death if AIF is not translocated into the nucleus, although it is released from mitochondria into the cytoplasm, as observed in the 3-MA pre-treatment group; and (iii) the nuclear translocation of AIF may be RIP3-dependent. So far, several other investigators have also reported that AIF participates in the process of programmed necrosis: the nuclear translocation of AIF in programmed necrosis of other cell types is prevented by Nec-1 or genetic knockout of RIP1 or RIP3^17^,^26^,^27^,^28^. These results demonstrate that RIP signaling acts upstream of the nuclear translocation of AIF, and our results further suggest that RIP3 combines with AIF in the cytoplasm, after which they are translocated into the nucleus together.

The role of mitochondria in apoptosis has been greatly clarified, but their contribution to programmed necrosis has not been clearly defined. It is possible that they function either upstream of necrosome formation or participate in the execution of necrosis^29^. When RIP3 is activated, it binds to and activates its substrate MLKL. Oligomerized MLKL translocates from the cytoplasm to mitochondrial and cell membranes to induce necroptosis^30^,^31^,^32^. The release of AIF from mitochondria was inhibited by Nec-1, but not fully by 3-MA in our study, suggesting that the release of AIF is RIP-dependent. Our findings indicate that mitochondria are involved in the execution of programmed necrosis, and AIF is the mediating molecule that links caspase-independent PCD with the programmed necrosis pathway.

## Conclusions

**Figure 6:**
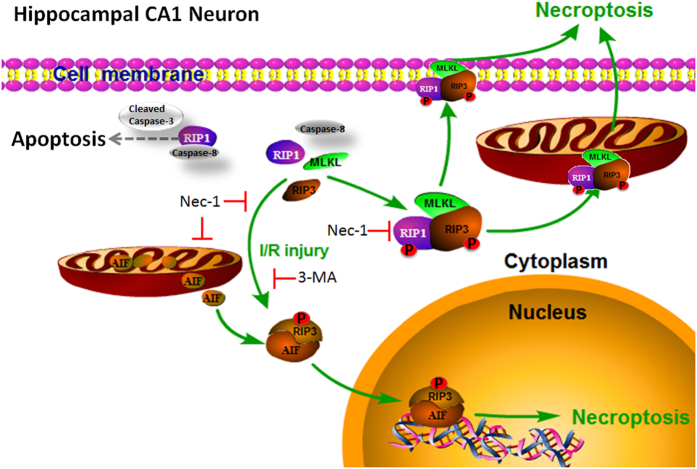
The signal pathway for hippocampal CA1 neuronal cell death induced by 20-min global cerebral I/R injury. RIP1 and RIP3 are activated (phosphorylated) and combine with each other after I/R injury. AIF is released from mitochondria and combines with RIP3 (perhaps phosphorylated RIP3) to form RIP3-AIF complexes. The RIP3-AIF complexes translocate into the nucleus resulting in chromatin condensation and DNA degradation, and then the neurons are triggered to undergo programmed necrosis. All of these changes after I/R injury are inhibited by pre-treatment with Nec-1 and 3-MA, except for the release of AIF from mitochondria in the 3-MA pre-treatment group. In neurons, the findings that caspase-8 expression was undetectable and caspase-3 was not activated indicate that caspase-dependent apoptosis is not involved in this process. Another necroptosis pathway in the cytoplasm induced by RIP1-RIP3-MLKL complexes, described by others, may also participate in this process^30^.

In most studies, RIP3 has been reported to be the key regulatory molecule in the cytoplasm in the necroptosis signal pathway. Here, we found another important signaling pathway of RIP3 in the nucleus in the neuronal programmed necrosis induced by I/R injury. Combining the present results with our previous studies^14^,^15^, the signaling pathway for hippocampal CA1 neuronal cell death induced by 20-min global cerebral I/R injury can be described as follows (Fig. 6). Elucidation of the death manner and mechanisms of neurons after I/R injury is helpful to the development of effective drugs and interventions in the future.

## Materials and Methods

### Animals

All of the experimental protocols were approved by the Ethics Committee on Experimental Animals at Zhejiang University and were carried out in accordance with the ARRIVE guidelines. Adult male Sprague-Dawley rats weighing 280–350 g were obtained from Zhejiang Experimental Animal Center. Five rats were placed in each cage under a 12-h light/dark cycle. The temperature was 24 ± 2 °C and the rats had free access to food and water.

### Global cerebral I/R injury model

Four-vessel occlusion for global cerebral ischemia with minor modification as described in our previous study was used^14^. Under 4% (w/v) choral hydrate (400 mg/kg) anesthesia, both vertebral arteries were permanently electro-cauterized and the bilateral common carotid arteries (CCAs) were freed from surrounding tissues. After closing the surgical incisions, rats were allowed to recover for 24 h. On the following day, anesthesia was induced with 4% isoflurane and the CCAs were occluded for 20 min with aneurysm clips to induce global cerebral ischemia, then the clips were removed for reperfusion. Rectal temperature was maintained at 37 ± 0.5 °C throughout the procedures. Rats were moved to an incubator to keep the proper temperature until they were fully awake. The mortality rate was calculated after reperfusion in each group.

### Intracerebroventricular injection and drug administration

3-Methyladenine (3-MA, Sigma-Aldrich, St. Louis, MO, USA. 600 nmol)^14^, necrostatin-1 (Nec-1, Merck, Kenilworth, NJ, USA. 1 μg)^15^, acetyl-L-aspartyl-L-methionyl-L-glutaminyl-L-aspart-1-al (Ac-DMQD-CHO, Enzo Biochem, New York, NY, US. 1 μg, 10 μg, or 100 μg)^33^, or vehicle was injected into the right cerebral ventricle 1 h prior to the induction of ischemia (anteroposterior −0.92; mediolateral 1.5; dorsoventral 3.5 mm) in a total volume of 5 μl at 0.5 μl/min. The needle was maintained in place for an additional 5 min before withdrawal to prevent fluid reflux.

### Tissue preparation

At 1, 2, 3, 7, or 30 days of reperfusion, rats were deeply anesthetized with 4% (w/v) choral hydrate (400 mg/kg) and perfused with saline at 4 °C, followed by 4% (w/v) paraformaldehyde in 0.1 mol/L phosphate-buffered saline (pH 7.4). Brains were removed and fixed over 2 days in paraformaldehyde. The post-fixed brains were embedded in paraffin.

### Hematoxylin and eosin (HE) and TUNEL staining

HE staining was performed on 3-μm coronal sections of hippocampus cut from paraffin-embedded blocks. Then the sections were passed through a decreasing alcohol gradient and stained with HE. After further immersion in ethanol and xylene, sections were mounted with resin and observed under a light microscope (Olympus IX81, Tokyo, Japan). After deparaffinization and rehydration, the sections were treated with 10 mmol/L protease K for 15 min, then TUNEL immunofluorescence staining was performed using an *in situ* apoptosis detection kit (Roche, Mannheim, Germany) according to the manufacturer’s instructions. The slides were then analyzed using a confocal microscope (Olympus FV1000). The number of surviving neurons, TUNEL-positive neurons, and total neurons in the CA1 layer per 1 mm length were counted. Then the neuronal survival and TUNEL-positive rates were calculated as the number of surviving or TUNEL-positive neurons/the total number of neurons.

### Immunofluorescence (IF) staining

After deparaffinization and rehydration, hippocampal sections were incubated in EDTA antigen revival buffer. After rinsing and blocking with 5% normal goat serum, sections were incubated overnight at 4 °C with one or two of the following antibodies: mouse anti-rat β-tubulin-III monoclonal (1:2000, Sigma, St. Louis, MO, USA), mouse anti-rat AIF monoclonal (1:200, Abcam, Cambridge, MA, USA), rabbit anti-rat RIP3 polyclonal (1:200, Biovision, Milpitas, CA, USA), mouse anti-rat Neun monoclonal (1:200, Abcam), rabbit anti-rat caspase-8 polyclonal (1:200, Abcam), rabbit anti-rat cleaved caspase-3 polyclonal (1:200, Cell Signal Technology, Danvers, MA, USA), rabbit anti-rat glial fibrillary acidic protein (GFAP) polyclonal (1:200, Cell Signal Technology), and donkey anti-rat ionized calcium-binding adaptor molecule-1 (Iba-1) polyclonal (1:200, Abcam). Anti-rabbit IgG: CY3 (1:200, Jackson, Baltimore, PA, USA), anti-donkey IgG: CY3 (1:200, Jackson), or anti-mouse IgG: FITC (1:200, Jackson) was added to each slide and incubated at 37 °C for 60 min. Since the GFAP and caspase-8 antibodies are from the same source (rabbit anti-rat), sections were incubated with GFAP antibody overnight and then with anti-rabbit IgG: FITC. After rinsing, the same sections were incubated with caspase-8 antibody overnight and then with anti-rabbit IgG: CY3 in sequence. All the sections were finally exposed to DAPI (Beyotime, Nanjing, China) to display nuclear changes. The slides were then read using a confocal microscope (Olympus, Tokyo, Japan).

### Western blot (WB)

Rat hippocampal CA1 tissues were rapidly isolated on ice at 2 days post-reperfusion. Total proteins were extracted and their concentration was determined by the Bradford method. We used a Cytoplasmic and Nuclear Extraction Kit (Invent Biotechnologies, Plymouth, MN, USA) to extract cytoplasmic and nuclear proteins. Forty microgram protein per lane were subjected to 15% sodium dodecyl sulfate-polyacrylamide gel electrophoresis for LC3 testing and 10% sodium dodecyl sulfate-polyacrylamide gel electrophoresis for AIF, RIP3, RIP1, and mixed lineage kinase domain-like polyclonal antibody (MLKL) testing. Then, the proteins were transferred to polyvinylidene fluoride membranes (Millipore, Billerica, MA, USA). The membrane was blocked with 5% non-fat milk and incubated with rabbit anti-rat LC3 polyclonal antibody (1:1000; Cell Signal Technology), rabbit anti-rat RIP1 monoclonal antibody (1:1000; Cell Signal Technology), mouse anti-rat AIF monoclonal antibody (1:500, Abcam), rabbit anti-rat RIP3 polyclonal antibody (1:200, Biovision), rabbit anti-rat MLKL (1:500, Abcam), or mouse anti-rat β-actin monoclonal antibody (1:1000; Sigma) at 4 °C overnight. After three washes, the membranes were incubated with HRP anti-mouse or HRP anti-rabbit secondary antibody (1:10000; Jackson) for 2 h. Bands were evaluated by the enhanced chemiluminescence method and were analyzed using ImageJ software.

### Immunoprecipitation (IP)

In brief, 500 μg protein was first pre-treated with rabbit anti-rat RIP3 polyclonal antibody (1:50, Biovision) for 4 h at 4 °C with agitation on a rotator. Protein A/G agarose (20 μL; Millipore) was added to each sample and incubated overnight at 4 °C. Then the mixture was precipitated by high-speed freezing centrifugation at 12000 rpm for 10 s. To remove non-specifically bound proteins, the sediment was washed three times with NP-40 buffer. Agarose-bound immunocomplexes were then released by denaturing solution in loading buffer. RIP3, RIP1, and AIF proteins in immunocomplex denaturing solution and total protein solution (for comparison) were analyzed by WB as above.

### Statistical analysis

The statistical analyses were performed using SPSS 16.0. All values are presented as the mean ± SD. One-way ANOVA with the Newman–Keuls post-test was used to compare the neuron counts in HE and TUNEL staining. Repeated measures and the nonparametric Friedman test were used to compare the relative WB band OD ratio values. *p* < 0.05 was considered to indicate a statistically significant difference.

## Additional Information

**How to cite this article**: Xu, Y. *et al*. RIP3 induces ischemic neuronal DNA degradation and programmed necrosis in rat *via* AIF. *Sci. Rep.* **6**, 29362; doi: 10.1038/srep29362 (2016).
